# Harnessing photochemical internalization with dual degradable nanoparticles for combinatorial photo–chemotherapy

**DOI:** 10.1038/ncomms4623

**Published:** 2014-04-07

**Authors:** George Pasparakis, Theodore Manouras, Maria Vamvakaki, Panagiotis Argitis

**Affiliations:** 1 Institute of Electronic Structure and Laser (IESL), Foundation for Research and Technology–Hellas (FORTH), PO Box 1527, 71110 Heraklion, Crete, Greece; 2 University College London, School of Pharmacy, 29-39 Brunswick Square, London WC1N 1AX, UK; 3 Institute of Nanoscience and Nanotechnology, NCSR Demokritos, 15310 Aghia Paraskevi, Attiki, Greece; 4 Department of Materials Science and Technology, University of Crete, 71003 Heraklion, Crete, Greece; 5 These authors contributed equally to this work

## Abstract

Light-controlled drug delivery systems constitute an appealing means to direct and confine drug release spatiotemporally at the site of interest with high specificity. However, the utilization of light-activatable systems is hampered by the lack of suitable drug carriers that respond sharply to visible light stimuli at clinically relevant wavelengths. Here, a new class of self-assembling, photo- and pH-degradable polymers of the polyacetal family is reported, which is combined with photochemical internalization to control the intracellular trafficking and release of anticancer compounds. The polymers are synthesized by simple and scalable chemistries and exhibit remarkably low photolysis rates at tunable wavelengths over a large range of the spectrum up to the visible and near infrared regime. The combinational pH and light mediated degradation facilitates increased therapeutic potency and specificity against model cancer cell lines *in vitro*. Increased cell death is achieved by the synergistic activity of nanoparticle-loaded anticancer compounds and reactive oxygen species accumulation in the cytosol by simultaneous activation of porphyrin molecules and particle photolysis.

## Abstract (web summary)

Photochemical internalisation is the process by which a laser source activates light sensitive compounds for cellular uptake. Here, the authors combine this technique with photo–chemo degradable polymers for the controlled uptake of chemotherapeutics into cancer cells showing increased cell death.

## Graphical abstract

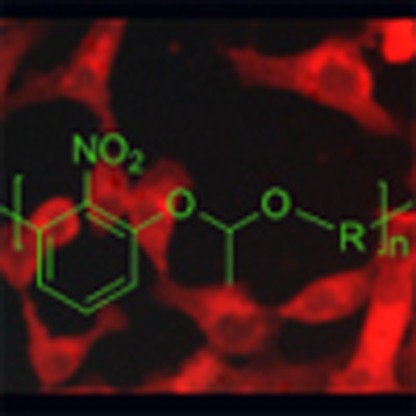

Modern nanomedicines promise to revolutionize cancer diagnoses and therapies by intervening to diseased tissues at the nanoscale and biomolecular level. The major advantage that nanoscale drug delivery systems (DDS) exhibit is the inherent passive tumour-targeting properties primarily derived by the enhanced permeation and retention effect attributed to the presence of leaky and ill-defined vasculature network and the lack of lymphatic drain^1^. Equally important is the possibility of active tumour targeting by the installation of recognition motifs on the surface of the nanoparticles (that is, specific cancer recognition markers, antibodies, and so on.)^2^,^3^. In addition, nanoparticulate DDS allow for simultaneous carriage of chemical substances such as anticancer agents and bio-imaging tags and have been proposed as an emerging platform in cancer theranostics^4^. The therapeutic benefit of polymeric DDS in tumour targeting through systemic administration has been demonstrated by numerous studies utilizing bioactive materials, including block copolymers, nanogels, and organic/inorganic hybrids, under various therapeutic scenarios such as against multi-drug resistant tumours^5^,^6^,^7^.

Although considering design parameters to mediate passive and/or active tumour targeting is of paramount importance to maximize the accumulation of nanoparticles (NPs) to tumour sites, intracellular barriers must also be considered to ensure drug release specificity at the organelles of interest^8^; effective endosomal escape is crucial to target the therapeutic cargo to specific cellular compartments. An appealing approach is the use of stimuli responsive DDS that exhibit sharp drug release changes in the presence of physiological gradients (that is, endosomal pH drop) or externally-controlled triggers such as temperature changes or light activation^9^,^10^. Light^11^, in particular, is a convenient means to externally activate DDS as it is generally safe, versatile – a wide range of wavelengths can be used – and can be delivered to nearly all parts of the body via catheterization or external sources^12^. Major obstacles, however, hamper the application of photocontrolled DDS in clinical use which include (i) the poor tissue penetration of certain wavelengths especially in the ultraviolet regime owing to extensive tissue absorbance and back scattering, (ii) the use of deep tissue penetrating wavelengths (in the near infrared regime) often requiring extensive focusing to achieve high (and potentially phototoxic) photon flux, which is not suitable for large tumour areas (see ref. 13 for a typical laser therapy protocol) and (iii) the lack of suitable drug carriers with high light-sensitivity^14^. In fact, the vast majority of studies on light-responsive DDS use ultraviolet A sources for activation (that is, wavelengths below 400 nm) and even when multi-photon excitation is employed, focused laser sources of high fluencies are used with limited working laser volume, up to a few micrometres only. Despite the substantial progress in light-responsive materials and biomolecular systems, and their applications in diverse areas, such as dynamic cell culture^15^,^16^,^17^, microfabrication^18^,^19^ and bioimaging^20^, the necessity of introducing new photochemistries that pragmatically address the aforementioned obstacles is of paramount importance for these technologies to realize their clinical potential in the context of modern therapeutic and diagnostic modalities.

To this end, we envisioned that photochemical internalization could serve as a model light–matter interaction motif in biological systems to challenge new photochemistries. Photochemical internalization (PI) is a conceptually powerful tool that was introduced to actively control the intracellular trafficking of anticancer agents via an exogenously applied optical stimulus, which allows for spatiotemporally-controlled drug release and trafficking. PI is applied by the codelivery of a bioactive agent (for example, a drug molecule) with a photosensitizer (for example, a porphyrin molecule) which upon cellular uptake is activated by a laser source^21^,^22^. In turn, the photosensitizer produces reactive oxygen species (ROS), which disrupt the membrane of the endosomal compartment and allow for effective escape of the drug molecules to the cytosol. PI has been successfully applied for the delivery of both high molecular weight (such as proteins and nucleic acids)^23^,^24^,^25^ and low molecular weight anticancer agents (such as camptothecin (CPT))^26^ under various therapeutic scenarios.

In the present study, we demonstrate for the first time, the application of PI in combinatorial photo–chemotherapy against cancer cells using a new class of dual degradable NPs coloaded with a potent chemotherapeutic anticancer compound (CPT) and a phototoxic drug (hematoporphyrin (HP)). Our system can be activated using visible, and potentially infrared wavelengths at very low doses delivered as unfocused pulse cues and exhibits simultaneous photo- and chemo-degradations, ideal for concerted light- and pH-controlled intracellular trafficking of drug cocktails, allowing for aggressive photo-induced cancer cell death. The proposed system induces enhanced cell death rates against cancer cells *in vitro*, owing to the concerted photo–chemotherapeutic potency of the drug cocktail over a wide surface area by laser activation using a clinically relevant light dosage protocol.

## Results

### Polymer synthesis and characterization

Considering the limiting factors that somewhat prevent the development of photosensitive nanomedicines (*vide supra*) we set out key design criteria to render the nanomaterials broadly addressable in the biomedical context: (i) simple chemistries were established so that the designed polymers are scalable and versatile for further optimization and formulation via self-assembly, (ii) we developed a mild photolysis strategy to allow for substantial red-shifting of the irradiation beam towards the visible and, if possible, the near infrared regime without compromising the laser working volume and (iii) we explored the possibility of loading drug cocktails (including poprhyrin molecules) to induce multimodal drug release against cancer cell lines via the photochemical internalization pathway.

We recently reported on a new class of polymers based on the ketal family^27^ that exhibits remarkably low ablation thresholds owing to their very low photolysis threshold, at least one order of magnitude lower compared with the heavily studied *o*-nitrobenzyl or other ultraviolet/visible labile materials^28^,^29^,^30^,^31^,^32^.

Acetals have a well established hydrolysis profile under the mildly acidic conditions (pH 5.5) found in the late endosome^33^,^34^. In an effort to introduce red-shifted photo-lability on the backbone of the polymer in the visible, a 2-nitroresorcinol comonomer was used.

**Figure 1:**
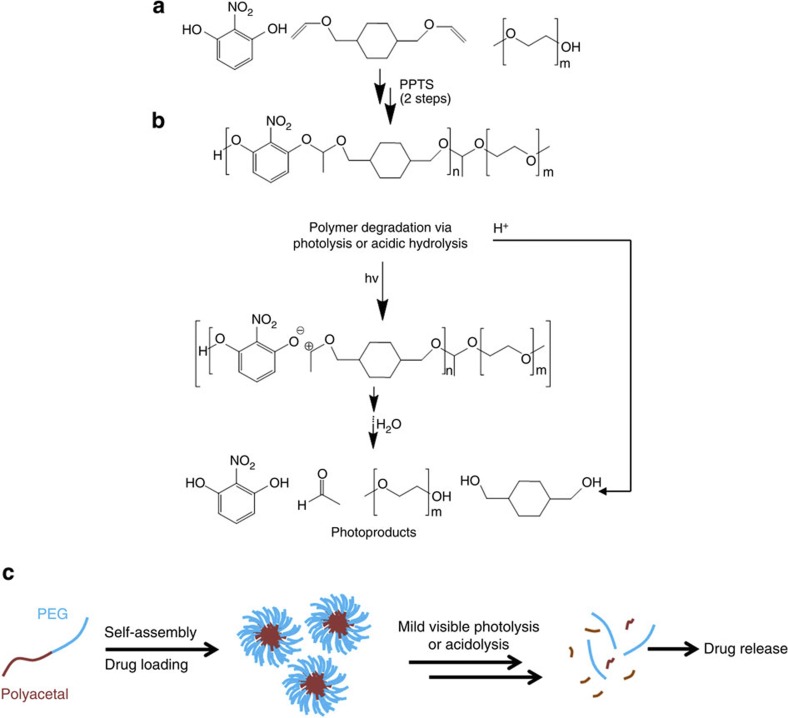
Synthesis, degradation and self-assembly properties of the block copolymer. (**a**) Synthetic route of the polycondensation reaction. For the precursor polymer, 2-nitroresorcinol and cyclohexyl divinyl ether were added in equimolar amounts with 1% PPTS (THF, N_2_, 12 h, 55% yield). For the block copolymer, 2 kDa PEG monohydroxy terminated was used with 1% PPTS (see experimental section for the detailed description), (**b**) the dual stimulus-mediated degradation of the polymers occurs either by ultraviolet/visible photolysis by single (365 nm), double (532 nm) or multi (1,064 nm, Supplementary Fig. 8) photon excitation, or acidolysis at mildly acidic pH (*ca*. 5.2) and (**c**) our proposed generalized strategy involves the self-assembly of the block copolymers in aqueous solutions, which allows for efficient loading of drug cocktails (that is, CPT and HP) that can be released by application of mild light irradiation and/or owing to the intracellular pH drop in the late endosomes upon cellular uptake.

The polymer was synthesized from commercially available synthons by a two-step acid catalysed polycondensation reaction of the 2-nitroresorcinol monomer with a divinyl ether derivative (first step), which was subsequently end-capped with poly(ethylene glycol) (PEG) (Fig. 1a) at the vinyl ends of the semitelechelic precursor polymer (second step). The precursor polymer was synthesized by mixing the two monomers at equimolar amounts in the presence of pyridinium *p*-toluenesulfonate (PPTS, 1%) as catalyst, and was isolated in a relatively good yield (55%) before characterization by proton nuclear magnetic resonance spectroscopy (^1^H NMR) (Supplementary Fig. 1) and gel permeation chromatography (GPC) (Supplementary Fig. 2). Then, the polymer was end-capped with monohydroxy terminated PEG (2 kDa) again catalysed by PPTS (1%) to afford the final block copolymer structure (Fig. 1a, and experimental section). The final product was isolated in good yield (*ca*. 80%) and characterized by GPC and ^1^H NMR (Supplementary Fig. 3 and Fig. 3a). The Mn of the polymer was found to be *ca*. 5,000 by GPC with a polydispersity index of 1.6, typical for polycondensation polymerizations. The final block copolymer consisted of a 2 kDa PEG block and a polyacetal block of *ca*. 9 acetal repeating units, which served as a self-assembled photo- and pH-labile block copolymer for multi-stimulus-activated drug release (Fig. 1c). The synthetic route that was followed involved the reaction of the divinyl and bis-hydroxyl monomers in a 1:1 molar ratio. The reaction kinetics of the polymerization is governed by step growth propagation^35^,^36^ where the starting monomers form dimers, which further react to form tetramers and so forth. Hence, the main product of the precursor polymer is a semitelechelic product with one vinyl and one hydroxyl end which, in theory, prevents the formation of triblock segments in the second step. Hence, further reaction with the PEG-hydroxyl should yield a di-block copolymer as the main product. In practice, the possibility of a triblock forming during the polymerization reaction cannot be neglected. However, from the GPC trace we do not see the formation of higher molecular weight products other than the final di-block copolymer and even if the triblock copolymer is present in a minute quantity it will also behave as a polymeric amphiphile and participate in the self-assembly process for the NP formation.

### Polymer photolysis at 365 nm

**Figure 2:**
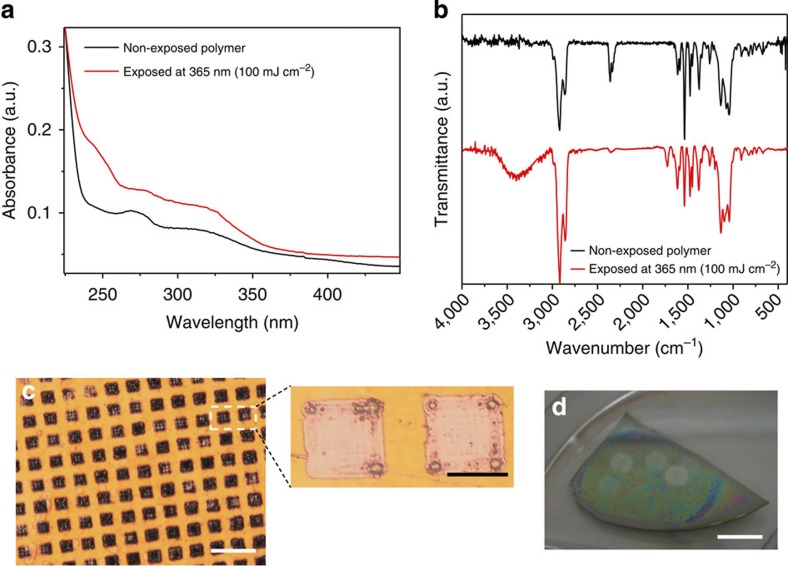
Polymer thin film photolysis studies. (**a**) Characteristic ultraviolet/visible and (**b**) FT-IR spectra of the non-exposed and exposed polymer films at 365 nm radiation demonstrating typical absorbance and transmittance peaks of the photoproducts formed, (**c**) optical microscopy image showing the pattern formation after laser ablation of the polymer film with a single laser pulse (532 nm, 20 mJ cm^−2^) by application of a TEM grid as photomask; simultaneous Fresnel patterns in the squares of the photomask shown as a close-up image. Scale bar, 200 μm (expansion scale bar, 50 μm). (**d**) Digital photograph of the ablated areas that can be seen with the naked eye on a piece of a polymer coated silicon wafer. Scale bar, 10 mm.

Photodegradation studies were first conducted in the solid phase by preparing thin polymer films on silicon substrates (400 nm film thickness). Upon light exposure with a Hg–Xe tool adjusted at 365 nm, effective photolysis of the films was evidenced by ultraviolet/visible and Fourier transform infrared (FT-IR) spectroscopy (Fig. 2a,b). Distinct absorption peaks appeared in the ultraviolet/visible spectrum of the irradiated sample, corresponding to the formation of carbonyl (250 nm) and nitroresorcinol photoproducts (330 nm). The photolysis mechanism was further corroborated by FT-IR spectroscopy where peaks at 3,400 cm^−1^ (broad) and 1,740 cm^−1^ appeared in the spectrum of the exposed sample, indicative of the formation of hydroxyl and carbonyl photoproducts. In addition, there was a noticeable change of the spectrum at 1,000–1,150 cm^−1^ assigned to the ether C-O-C stretching, overlapping with the cleavage of the acetal O-C-O bonds. The photolysis process is hypothesized to be triggered by direct zwitterionic cleavage of the acetal proton followed by acid hydrolysis and formation of the starting 2-nitroresorcinol, acetaldehyde and cyclohexyl di-alcohol byproducts (Fig. 1b).

### Photolysis of acetal (Ac1) and photoproduct characterization

To further elucidate the photolysis mechanism, we synthesized a low molecular weight compound resembling the repeating unit of the polymer, in an effort to characterize the photolysis products by mass spectrometry. The molecule Ac1 (Supplementary Fig. 4) was subjected to complete photodegradation by ultraviolet irradiation at 365 nm and the photoproducts formed were studied by ^1^H NMR and gas chromatography–mass spectrometry (GC–MS). From the ^1^H NMR spectrum, it was possible to clearly trace the formation of acetaldehyde, 2-nitrophenol and the aliphatic alcohol derivative, which are the main photoproducts, along with the complete disappearance of the acetal proton as a result of the quantitative photolysis (Supplementary Fig. 5). The possible formation of carbonyl-rich photoproducts was evident in the 1.5–2.1 p.p.m. range of the ^1^H NMR spectrum, which could be confirmed by GC–MS (Supplementary Figs 5 and 6). The latter confirmed the formation of the 2-nitrophenol and the aliphatic di-alcohol, however it was not possible to trace acetaldehyde, presumably owing to its low molecular weight, below the detection limit of the instrument. It should be noted that the fact that 2-nitrophenol is unambiguously traced by both ^1^H NMR and GC–MS, excludes the possibility of formation of other nitroso derivatives, which in turn confirms our proposed mechanism of acetal photolysis involving the zwitterion^37^ intermediate formation as proposed in Fig. 1b.

We also used Ac1 as a model molecular probe to monitor the photolysis kinetics by following the gradual diminution of the acetal proton peak as a function of the irradiation time (Supplementary Fig. 7); it is apparent that the photolysis progresses by a non-linear parabolic rate and reaches 100% at a total irradiation dose of 1 J. To sum up, the main photoproducts comprise the formation of acetaldehyde, 2-nitrophenol and cyclohexyl di-alcohol products; carbonyl-rich photoproducts are also evident but these should exist at minute quantities and are not uncommon in photolysis studies.

### Two-photon polymer photolysis

Encouraged by these results, we studied the two-photon photolysis properties of the polymer in the solid and liquid phase. The second harmonic of a neodymium-doped yttrium aluminium garnet (Nd:YAG) laser (532 nm, 7 ns pulse duration) was chosen for the red-shifted photolysis experiments as it is currently used in the clinic and exhibits adequate tissue penetration depth^38^. Nearly complete photolysis of the polymer film was observed after laser irradiation with a single unfocused pulse with energy fluence as low as 20 mJ cm^−2^ (Fig. 2c). The film ablation was effective across the whole area of irradiation, which was evident from the pattern formation by application of a photomask (Fig. 2c and d). Characteristic Fresnel diffraction patterns were observed on the residual debris indicating that the pulse energy was close to the ablation threshold. It should be noted that near field diffraction is found in multi-photon excitation processes using unfocused laser sources^39^.

**Figure 3:**
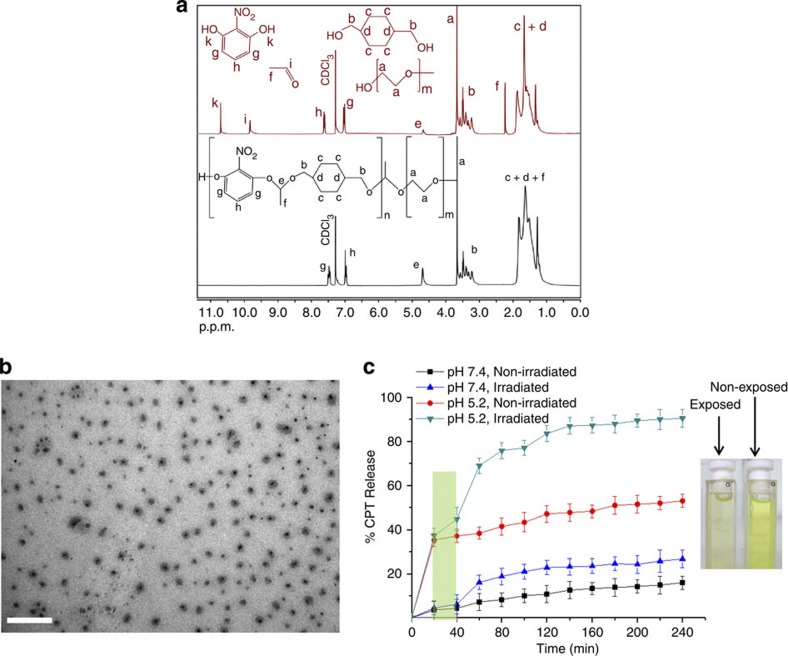
Polymer and NP photolysis in solution and light/pH-controlled CPT release. (**a**) ^1^H NMR spectra of the initial polymer solution in *d*-chloroform (non-exposed) (in black) with the corresponding spectrum (in red) of the laser-irradiated sample showing characteristic diminution of the acetal proton (peak e) and the appearance of acetaldehyde (peaks i and f) and 2-nitroresorcinol (peak k) protons, (**b**) TEM microphotograph of polyacetal nanoparticles and (**c**) % CPT release from NPs at different pH and laser irradiation conditions (the green stripe represents the irradiation interval), and digital photograph of laser-exposed and non-exposed polymer solution. Scale bar, 500 nm.

The photolysis of the polymer in solution was conducted in deuterated chloroform to allow for direct monitoring of the photodegradation by ^1^H NMR. The polymer solution was irradiated with 20 mJ cm^−2^ pulses for 10 s with frequency of 5 Hz and a total energy dose adjusted at 1 J so that the photolysis protocol is clinically valid for photodynamic therapy, but also to maximize the potency of the photolysis process. The NMR spectrum of the laser-exposed sample clearly showed the formation of acetaldehyde and 2-nitroresorcinol, and the simultaneous decrease of the acetal proton peak at 4.5 p.p.m. as a result of the photocleavage of the photolabile polymer block (Fig. 3a). The photolysis process was *ca*. 70% complete judging from the relative integrals of the acetal peaks with respect to the non-exposed sample (see experimental section for details). We attribute the incomplete photodegradation to the absence of water in the sample, which is necessary for more effective photodegradation and to the inhomogeneous irradiation of the sample by the laser beam. Nevertheless, the effect of laser photolysis was visible with the naked eye as the polymer solution gradually lost its vivid yellow colour during laser irradiation (Fig. 3c).

### Drug release studies

Having established the photolysis conditions at 532 nm, drug release studies were performed to optimize the conditions for PI. The polymer formed spherical nanoparticles in water with mean diameter of 190 nm determined by dynamic light scattering (DLS) (equation (2), Supplementary Fig. 9). The morphology of the nanoparticles was examined by transmission electron microscopy (TEM), which confirmed their spherical shape and their relatively uniform size distribution (Fig. 3b). Close inspection of the nanoparticles revealed an aromatic-rich core indicative of the strong electron-rich signal in the TEM micrographs (darker areas), whereas the hydrophilic PEG residues appeared in lighter grey colour. The nanoparticles were found to be stable for weeks in slightly alkaline solutions (that is, in PBS pH 7.4 to prevent acetal hydrolysis).

CPT was combined with HP to induce multimodal-type cell death and to apply PI as a drug codelivery modality activated by concerted pH and light stimuli (Fig. 1c). HP is a widely used photosensitizer that produces ROS on light exposure of various wavelengths owing to the broad-absorbing band of porphyrin molecules. CPT is a potent alkaloid that interacts with DNA and topoisomerase I and leads to apoptotic cell death. CPT is hydrophobic and could be incorporated in the lipophilic core of the NPs; in addition, CPT encapsulation limits the possibility of possible opening of the lactone ring, which could compromise its activity. HP is also hydrophobic and can be loaded in the cores of the NPs, however, its water solubility can be tuned by varying the pH owing to the presence of the carboxyl groups adjacent to the porphyrin ring. HP and CPT have distinct emission profiles when excited at 350 nm (Supplementary Fig. 10), which was convenient to quantify the drug loading (equation (1)) capacity of the nanoparticles (17% and 11% for CPT and HP, respectively). CPT was used as a reference compound to monitor the drug release rates as a function of pH variation and laser irradiation.

Relatively low drug leakage from the NPs was recorded at alkaline pH, which did not exceed 15% of the total-loaded CPT after 4 h, which was indicative of the acetal bonds’ resistance to hydrolytic cleavage (Fig. 3c). Under the same conditions, laser irradiation resulted in nearly double CPT release (26%) as a result of the partial NPs’ photolysis.

Conversely, the drug release rate was significantly more pronounced when the pH was lowered to 5.2, which is the pH found in the late endosome where PI takes place. The total CPT release reached 52% in the non-irradiated sample as a result of the hydrolysis of the NPs. The CPT release was dramatically increased upon laser exposure and exceeded 90% as a result of the combinational NPs’ photo-acido-lysis.

It is also apparent that the acidic samples exhibit an initial burst-type drug release in the first 20 min and then the slope of the rate of CPT release is significantly lower (Fig. 3c). This is attributed to the fact that the polymer hydrolysis should be initiated from the acetal moieties adjacent to the hydrophilic PEG block, which enhances their solvation and hence their susceptibility to acid hydrolysis. Then the hydrolysis rate should be governed by the less solvated NP core and is subjected to acidic hydrolysis at a lower rate, which is reflected to the CPT-release profile.

### Preliminary cytotoxicity studies

Cytotoxicity studies were performed by collecting CPT samples at the end of the drug release experiments (after 4 h, Fig. 3c) to probe the effect of diffusive- or stimulus-mediated (pH and/or light) CPT release on HeLa cells (Supplementary Fig. 11). The minute CPT leakage after 4 h induced limited cell death in both alkaline, non-irradiated (8%) and irradiated (12%) samples; the cell death rates were found to be statistically insignificant and hence we presume that the CPT released from these samples should be well below the IC_50_ onset for effective cytotoxicity. The death rates significantly increased in the case of the acidic samples (27% and 54%, for the non-irradiated and irradiated sample, respectively) as a result of the more pronounced CPT release owing to the acidolysis (or combined acido-photo-lysis) of the NPs, which resulted in 52 and 91% release of total-loaded CPT (Fig. 3c). These results underline the fact that the minor CPT leakage from the dormant NPs (alkaline, non-irradiated) exerts very low cell death rates compared with the activated NPs (acidic and irradiated) and hence support our drug loading and release methodology in the proposed context of laser-mediated intracellular activation of the NPs.

### Cellular uptake and photochemical internalization

Similar experimental conditions were applied for the *in vitro* studies to prove the concept of using chemo- photo-degradable NPs in PI protocols.

**Figure 4:**
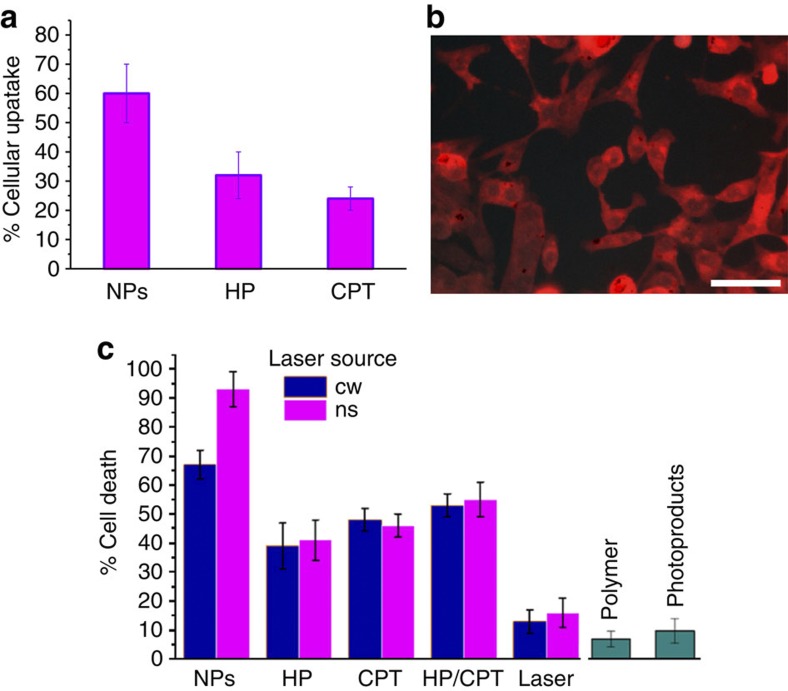
NPs cell uptake and cytotoxicity studies. (**a**) Increased cellular uptake of the NPs compared with the non-encapsulated drugs, (**b**) characteristic fluorescence microscopy image of HeLa cells incubated with drug-loaded NPs, the red filter has been used to track the distinct red emission profile of HP in the cytoplasm and (**c**) cell death rates in respect to irradiation using different laser sources and control samples (namely, free HP or CPT, free CPT/HP and lasers alone). Scale bar, 100 μm.

Subsequently, CPT- and HP-loaded NPs were incubated with HeLa cells for 4 h and their cellular uptake using fluorescence microscopy were examined. The cellular uptake was significantly higher (62%) in the case of the drugs being loaded in NPs than the non-encapsulated HP and CPT controls (35% and 29%, respectively, Fig. 4a). This is attributed to the hydrophobic nature of both CPT and HP molecules, which results in non-specific binding to the proteins of the culture medium and in turn compromises the cytoplasmic entrance by the lowering of their effective concentration for cellular uptake. Additionally, it is well-documented that nanoparticles in the size range of 100–200 nm (as in this study), are preferably uptaken by the endosomal pathway via clathrin-dependent endocytosis, which is a relatively fast process (compared with the macropinocytotic pathway)^40^,^41^. The cellular uptake of the NPs was visible under the fluorescence microscope by tracking the strong red emission of HP (Fig. 4b) via excitation of the Soret band at 400 nm.

Finally, the resazurin assay was performed to evaluate the possible toxicity of the polymer and its photoproducts as well as the potency of laser irradiation in cell death of the drug-loaded NPs against various controls. The polymer was found to exhibit minimum cell toxicity when tested in similar concentrations to the actual drug-loaded formulation, but more importantly, the photolysed material also showed minute toxic effects *in vitro* (Fig. 4c). Laser irradiation studies were performed by using continuous wave (CW) and pulsed lasers to rule out possible effects of single- or multi-photon phototoxic events but also to probe the role of particle photodegradation in the overall cell death mechanism. The total dose delivered to cells was determined to be 1 J and was delivered by unfocused pulsed cues with an effective area of 0.7 cm^2^ to maximize the effective laser area. Strikingly, the cell death rate was considerably higher (exceeded 93%, Fig. 4c) in the case of NPs irradiated with the YAG laser at 532 nm compared with all other controls, namely, CPT or HP alone, and CPT/HP combined but not NP loaded. Also, minor death rates, presumably owing to phototoxic events, were observed by using lasers only without the presence of drugs under the followed irradiation protocol. Interestingly, the type of laser does not seem to have a statistically significant effect on any of the controls used except for the drug-loaded NPs were the particle photolysis seems to enhance the cell death rates by 30% compared with the CW-irradiated NPs (65%) where no synergistic particle photolysis takes place (Fig. 4c).

**Figure 5:**
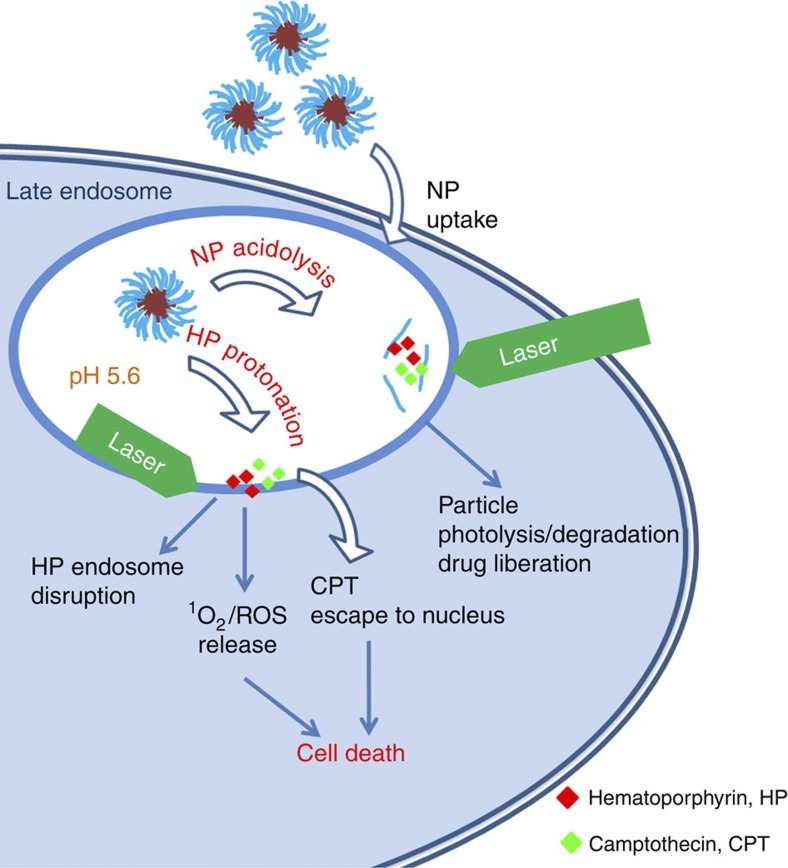
Photochemical internalization augmented by NPs photo–chemo-lysis. The proposed mechanism of action involves the cellular uptake of the drug-loaded NPs followed by synergistic photo–chemo-degradation, which leads to enhanced drug liberation and endosome photo-disruption via HP activation and hydrophobization; cell death is augmented by the combination of the CPT-topoisomerase I interaction and the phototoxic pathway via ROS accumulation in the cytoplasm.

It is noteworthy to analyse the mechanism of action of the system developed to understand the potency of its cytotoxicity (Fig. 5). First, the NP compartment allows for simultaneous delivery of two active agents, CPT and HP, which are in close proximity during the delivery events. NPs are presumably uptaken by cells via the endocytosis pathway and end up in the late endosome. The gradual acidification of the late endosomal compartment leads to partial hydrolysis of the NPs, which is augmented by the laser photolysis. The particle degradation is followed by drug liberation and association of CPT and HP with the endosomal membrane. In particular, protonation of the HP molecules (two pK values are found in the pH range 2.9–11.3, namely pK 5.7 and 8.2 (ref. 42)) in the acidic environment increases their lipophilicity leading to more pronounced interaction with the endosomal bilayer. Then, laser activation results in singlet oxygen production via photosensitization of the HP molecules, which in turn disrupt the endosomal membrane, assisting CPT molecules to escape from the compartment and trigger cell death pathways. In essence, the cell death is concerted by the synergistic excessive ROS accumulation and the enhanced CPT release from the endosome during laser treatment. However, it is detrimental to ensure that both drugs are in close proximity to exert amplified cell death rate, which apparently, is not observed when the two drugs are delivered simultaneously but without being loaded in NPs (Fig. 4c).

It should be noted that secondary mechanism pathways of action may exist; for example, it is possible that a small fraction of particles are photolysed before they reach the acidic environment of the late endosome (that is, in lysosomes), but still contribute to the ROS generation and CPT liberation. Therefore, to fully deconvolute the role of pH versus light irradiation on the intracellular trafficking of the drug cocktail, a detailed biophysical study will be required in the future to track the NPs’ intracellular pathways via confocal microscopy.

## Discussion

Photodegradable materials^29^ have only recently emerged as a new means to exogenously manipulate living matter and control biological processes. Notable applications include dynamic cell culture, scaffold biofabrication, cell patterning technologies and biomolecule microarray construction. Although, the chemical repertoire for caging (and photo-unmasking) small molecules has been extensive^11^, the application of such photochemistries in drug delivery technologies has been rather limited. Ultraviolet light sources provide poor tissue penetration depth and usually multi-photon excitation requires highly focused laser beams that somewhat limit the clinical potential of these systems. Our approach involves the use of novel-type photolabile chromophores that absorb in the visible regime and comprise acetals as the labile moieties that exhibit very low ablation threshold, which is significantly lower than polyesters, styrenics and polytriazenes, among other materials, and is the only class of polymers with such low photolysis thresholds with visible irradiation^43^. Our strategy, allows for facile tuning of the irradiation wavelength depending on the adjacent chromophore to the acetal unit. Hence the photolysis process can be triggered by using either ultraviolet (193 and 248 nm)^27^, ultraviolet A (365 nm), visible (532 nm) and near infrared (1,064 nm, Supplementary Fig. 8) light sources either by single, double, or multi-photon excitation. Furthermore, the concerted photo and chemo-lability of the polymers render them ideal carriers to apply PI protocols to overcome biological barriers and target specific cellular organelles. In addition, the polymers developed in this study serve as excellent multi-drug carriers as they form nanoparticles with aromatic-rich cores, which can safely host combinations of hydrophobic drugs and photosensitizers via π-π stacking and/or hydrophobic interactions with minute leakage. Drug coencapsulation in nanoparticulate matrices constitutes an alternative method to deliver drug and biologics using PI and complements direct conjugation methods where the drug of interest is covalently linked with a photosensitizer.

It is envisaged that the combination of PI with photodynamic therapy will circumvent the inherent difficulties in certain tumour types where drug resistance limits the therapeutic outcome of classic chemotherapy. In this context, the development of suitable drug carriers and chemistry motifs to deliver drug cocktails that are exogenously activated at specific diseased sites at large tissue areas is of paramount clinical importance. The extremely low ablation threshold of these materials, allows for significant widening of the laser working volume to the millimetre or even the centimetre scale which opens new possibilities in medically-related photochemistries to be applied at the macroscale, for example, in laser-mediated tumour ablation, and laser/image-guided drug delivery and surgery. In addition, the main-chain scission type of combined polymer photo-acido-lysis leads to the formation of non-toxic low molecular weight photoproducts, which render the system appealing from a pharmacological viewpoint.

In a wider context, the formulation of multifunctional nanomedicines (including multiresponsive DDS) for therapy and imaging poses tremendous challenges in terms of therapeutic value in respect to manufacturing cost and toxicity issues^4^. One approach to circumvent these limitations is the introduction of DDS constructed by a limited number of building blocks that exert multiple biological/therapeutic activities via rational design principles. For example, in the present study we demonstrated that by a dual stimulus mechanism, it is possible to induce effective PI-based drug delivery via the rational coupling of the nanocarriers’ properties with the drug cocktail. In essence, the proposed DDS responds in four different, but synergistic, mechanisms triggered by two stimuli: the pH drop induces simultaneous polymer degradation and change in the lipophilicity of HP, while the application of laser irradiation induces polymer photolysis and ROS production to mediate PI.

We feel that the materials developed in this study will certainly open up new avenues in the development of such combinational therapeutic modalities and will fuel the field of light-controlled DDS to complement the landscape of exogenously-activated DDS, which are currently limited to thermal (that is, Thermodox^44^) or magnetic (that is, Magforce^45^) means of activation.

In conclusion, we have developed new materials that exhibit mild and tunable photolytic cleavage under clinically relevant conditions. We demonstrated the use of dual photo- and chemo-degradable NPs in photochemical internalization-mediated delivery of cytotoxic agents and proved the concept of enhanced cytotoxicity against cancer cell lines *in vitro*. The block copolymer NPs were synthetized using scalable synthetic strategies and we managed to red-shift the photolysis wavelength to the visible regime but also to keep the photolysis threshold at low fluencies. Polyacetals were subjected to single, double and multi-photon laser photolysis which, in combination with the acidic degradation, was shown to simultaneously control the intracellular activation and release of drug cocktails and mediate amplified cancer cell cytotoxicity. Finally, it is encouraging that the developed chemistry is also applicable in the infrared regime which, after further optimization of the labile chromophores, will pave the way for *in vivo* studies.

## Methods

### Materials and instrumentation

All solvents and reagents were purchased from Sigma or Alfa Aesar and were of analytical or HPLC grade. Gel permeation chromatography was carried out using a Thermo Scientific Spectrasystem P1000 isocratic pump equipped with a ultraviolet and refractive index detector and mixed D and mixed E columns. The molecular weights of the polymers were determined by the universal calibration method using polystyrene standards. Tetrahydrofuran (THF) at a flow rate of 1 ml min^−1^ was used as the mobile phase. NMR studies were carried out on a Bruker Advance DPX 300 NMR Spectrometer (300 MHz). Ultraviolet/visible spectroscopy was performed using a Perkin-Elmer spectrophotometer. FT-IR studies were carried out using a Bruker, Tensor 27 spectrometer. An Oriel 500 W Hg-Xe lamp was used for the photodegradation studies. A Nd:YAG nanosecond laser (7 ns pulse duration) was used for the cell photolysis experiments. Optical microscopy was performed on a Leica DMRM microscope equipped with a digital camera connected to a PC. GC–MS was carried out on a Shimadzu GC–MS-QP2010 Ultra spectrometer.

### Precursor polymer synthesis

In a typical polymerization reaction, 2 g (0.013 mol) of 2-nitroresorcinol were dissolved in 20 ml dry THF under magnetic stirring in a 50 ml round bottom flask sealed with a rubber septum. A THF aliquot of 1,4 cyclohexanedimethanol divinyl ether (2.77 ml, 0.013 mol) was transferred in the flask through a glass syringe. The polymerization reaction was initiated by adding 1% PPTS (0.033 g, 0.13 mmol, molar equivalent with respect to the monomers) in the THF solution, which was left under stirring for 12 h in the dark. The reaction was stopped by adding 1–2 drops of triethylamine to quench the catalyst. The polymer was isolated by precipitation in hexane followed by extensive drying under vacuum to constant weight. The polymer was obtained as a pale yellow oil (yield 55%).

### Block copolymer synthesis

The precursor polymer (2,800 MW) was reacted with a monoydroxy PEG derivative of 2,000 Da as follows. 2 g PEG were dissolved in dry THF (10 ml) and then transferred to a freshly-prepared polymer solution (10 ml THF, 2.8 g precursor polymer). 1% PPTS with respect to the total polymer moles was added to initiate the reaction, which was left under stirring in the dark for 24 h. The reaction was stopped by adding 1–2 drops of triethylamine. The final product was collected by precipitation in hexane followed by extensive drying under vacuum to constant weight. Extensive dialysis against THF was performed to isolate the block copolymer from the unreacted PEG and lower molecular weight byproducts (Supplementary Fig. 1). A regenerated cellulose membrane with molecular weight cut-off (MWCO) 3,500 Da was used throughout the purification process before characterization with GPC and NMR (Supplementary Fig. 2).

### Synthesis of 2-nitrobenzene-2-ethylhexyl methyl Ac1

The acetal-containing compound was synthesized by the electrophilic addition of 2-nitrophenol (1.2 g, 8.64 mmol) and 2-ethylhexyl vinyl ether (1.38 ml, 7.2 mmol) in dry THF (10 ml). The reaction was initiated by the addition of PPTS (18 mg, 0.072 mmol) and was left at 50 °C for 24 h. The reaction was stopped by the addition 0.1 ml of triethylamine and the THF was removed using a rotary evaporator. The reaction mixture was then transferred to dichloromethane and the product was isolated by washing five times with water to remove the excess of 2-nitrophenol and the catalyst. Removal of the dichloromethane afforded the final product as a yellow oil (1.4 g, yield 66%), which was then characterized by ^1^H NMR and GC–MS along with its photoproducts.

### Polymer film preparation and laser irradiation

Thin polymer films were prepared by spin coating a polymer solution (5% in THF) on silicon wafers (1,000 r.p.m., 1 min). The polymer films were extensively dried using N_2_ steam and stored in the dark before use. The film thickness was measured by ellipsometry and was found to be *ca*. 400 nm. The polymer films were irradiated either directly or by application of a TEM grid (50 μm square patterns) as a photomask.

### Photolysis of Ac1 and characterization of the photoproducts

Ac1 was dissolved in deuterated dimethyl sulfoxide (5 mg ml^−1^) and subjected to irradiation at 365 nm. The ^1^H NMR spectrum was recorded at predetermined time intervals to record the kinetics of the photolysis process (Supplementary Figs 5 and 7). The photoproducts were also characterized by GC–MS (Supplementary Fig. 6).

### Nanoparticles preparation

Polymer nanoparticles were prepared by adding dropwise a polymer solution in THF (5%, 10 ml) to 200 ml water or phosphate-buffered saline (PBS) (pH 7.4, 100 mM). THF was completely removed under vacuum before storing the NPs in the dark at −18 °C.

### Drug loading and release

A polymer/drug solution was prepared in methanol (20 ml, 1 *g* polymer, 50 mg HP and 50 mg CPT), which was left to form a thin film on the wall of a round bottom flask by letting the solvent to evaporate slowly. Then, PBS (50 ml, pH 7.4, 100 mM) was slowly added and left under gentle stirring for 2 h in the dark. The NPs suspension was transferred in a cellulose dialysis bag and was dialysed against excess PBS (1 l) for 8 h to remove the unloaded drugs. The total drug loading was calculated based on equation (1).


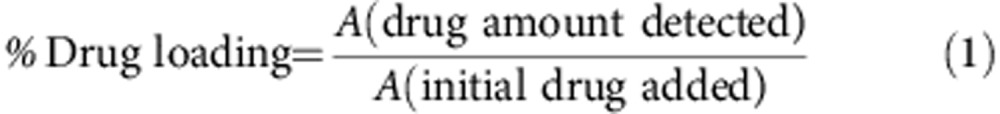


where A is the emission value obtained from fluorescence spectroscopy of known sample concentrations compared with standard calibration curve for HP and CPT. Excitation at 350 nm was used throughout the experiments as at this wavelength both drugs exhibit distinct emission bands and are easy to trace (Supplementary Fig. 10).

The dialysis method was used for the drug release studies; a drug-loaded NP suspension in PBS (10 ml, pH 7.4) was dialysed against either acidic (500 ml, pH 5.2) or basic buffer (500 ml, pH 7.4) to probe the effect of particle hydrolysis. 1 ml aliquots were collected followed by subsequent buffer replenishing at predetermined time intervals and the fluorescence emission was measured to construct the drug release profiles. The NP suspension was kept under constant gentle stirring. Laser irradiation was conducted by irradiating the NP suspension directly from above without disturbing the sampling setup. The drug release profiles were normalized against the total CPT loaded for each sample.

A preliminary cell death study was conducted to determine the possible cytotoxicity of the CPT released after 4 h. CPT samples were collected and the CPT concentration was diluted to match the volume of the aliquots used in the control cell death assays (Supplementary Fig. 11).

### DLS

A dilute NP suspension in water was freshly prepared (0.5 mg ml^−1^) for the DLS measurements. DLS was carried out on a custom-made system. The light source was an Adlas DPY 315 II Nd:YAG laser (532 nm), polarized by two Glan-Thomson polarizers (Halle, Berlin) placed before and after the sample. A colourless lens was used to focus the beam onto the sample with a focal length of 200 mm. The scattered light was collected by an optical detector utilizing a 2F/2F system. Two pinholes were used before the photomultiplier (Thorn EMI) to define the scattering volume. The photon correlation was performed by an ALV-5000/E photon correlator. The thermostatted sample cell was placed on a motor-driven precision goniometer, which enabled the photomultiplier detector to be moved accurately from 11° to 150° scattering angle. The sample was placed in a toluene bath and the temperature was monitored by a thermostat. The resulting correlation functions were fitted with a stretched exponential function, Kohlrausch–Williams–Watts from which the relaxation time was derived, and allowed to calculate the diffusion coefficient of the scatterer. The hydrodynamic diameters were calculated from the Stokes–Einstein equation (2).


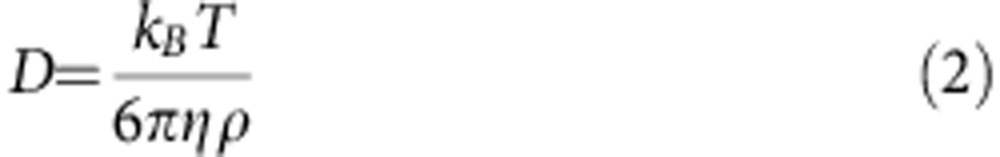


where *D* is the diffusion coefficient, *k*_*B*_ is Boltzmann’s constant, *T* is the absolute temperature, *η* is the viscosity of the solvent and *ρ* the hydrodynamic radius of the spherical particle.

### TEM

A drop of the diluted sample was placed on a carbon-coated copper grid (400 Mesh) and was first gently purged with a N_2_ steam and then left to dry in the dark overnight. A JEOL JEM-2100 instrument at an electron accelerating voltage of 80 kV was employed for the measurements.

### Cell culture and NP uptake

HeLa cells (purchased from ATCC) were suspended to a concentration of 10^5^ cells ml^−1^ in Dulbecco’s modified Eagle’s medium with 10% foetal bovine serum, and 1% antibiotic solution (GIBCO, Invitrogen, Karlsruhe, Germany). The cell suspension was transferred in a polystyrene-96-well plate (180 μl in each well) and cultured at 37 °C and 5% CO_2_. The cells were visually examined regularly by optical microscopy and the culture was stopped after 48 h. The cells were incubated with NPs by adding 20 μl of PBS NP suspension (2 mg ml^−1^, loaded with HP and CPT) to a total volume of 200 μl. After incubating for 4 h, the culture medium was replaced with fresh PBS and the cellular uptake was measured on a plate reader. Equimolar amounts of free HP and CPT as determined from the drug loading studies were used as control experiments to probe the effect of drug encapsulation on the cellular uptake. The uptake studies were performed in triplicate.

### Polymer and photoproducts cytotoxicity

In the previously described protocol, suitable control experiments were conducted to address the cytotoxicity of the synthesized polymer and its photoproducts. The cells were suspended with the polymer by adding 20 μl of a PBS polymer solution (2 mg ml^−1^). The cells were also incubated with the polymer photoproducts as collected by a previously laser-irradiated PBS polymer solution (2 mg ml^−1^, irradiated with a total dose of 1 J, which was delivered at a frequency of 2 Hz and fluency of 20 mJ cm^−2^). Subsequently, a live/dead cell counting assay was performed to assess the cytotoxicity of the samples as described below.

### Laser irradiation

After the cell uptake studies the six-well cell-containing plate was transferred to a x-y positioning stage for laser irradiation. The cells were irradiated with a Nd:YAG laser (532 nm) with a total dose of 1 J, which was delivered as unfocused pulses with effective area of 0.7 cm^2^ at a frequency of 2 Hz and fluency of 20 mJ cm^−2^. This protocol was used as it allows for effective particle photolysis and also permits adequate heat dissipation between pulses to avoid photothermal cell death. The same setup was used for the control experiments using the CW laser source at the same total dose.

Note: Although we did perform irradiation studies at 1,064 nm (Supplementary Fig. 8) we had to increase the pulse fluence to 50 mJ cm^−2^ to probe effective particle photolysis, however, photothermal cell death was more pronounced and hence more difficult to deconvolute from the overall photochemical internalization mechanism. We are currently exploring other chromophores adjacent to the acetals that lower the ablation threshold at 1,064 nm–this research is currently ongoing and the data will appear in another study.

### Live/dead assay

The Vybrant cytotoxicity assay (Molecular Probes) was used as it allows for accurate viability rate determination at low cell numbers (down to 500 cells according to the manufacturer) and does not interfere with the emission bands of HP ad CPT. We followed the manufacturer’s protocol to perform the resazurin assay, which is described in detail elsewhere^27^. The assay was performed in triplicate to ensure statistical validity. The unpaired Student’s *t*-test was used to test the statistical significance of the viability rates.

## Author contributions

G.P. and T.M. conceived the concept, performed the experiments and analysed the data. M.V. and P.A. overviewed the project and commented on the results. G.P. wrote the manuscript with inputs from all authors.

## Additional information

**How to cite this article:** Pasparakis, G. *et al.* Harnessing photochemical internalization with dual degradable nanoparticles for combinatorial photo–chemotherapy. *Nat. Commun.* 5:3623 doi: 10.1038/ncomms4623 (2014).

## Supplementary Material

Supplementary Information Supplementary Figures 1-11
