# Supplementary material for: Harnessing photochemical internalization with dual degradable nanoparticles for combinatorial photo–chemotherapy
Source: Nat Commun. 2014 Apr 7;5:3623. doi: 10.1038/ncomms4623 (PMC3988806; doi:10.1038/ncomms4623)
Supplement: Supplementary Information — Supplementary Figures 1-11 [file ncomms4623-s1.pdf]

Supplementary Figures

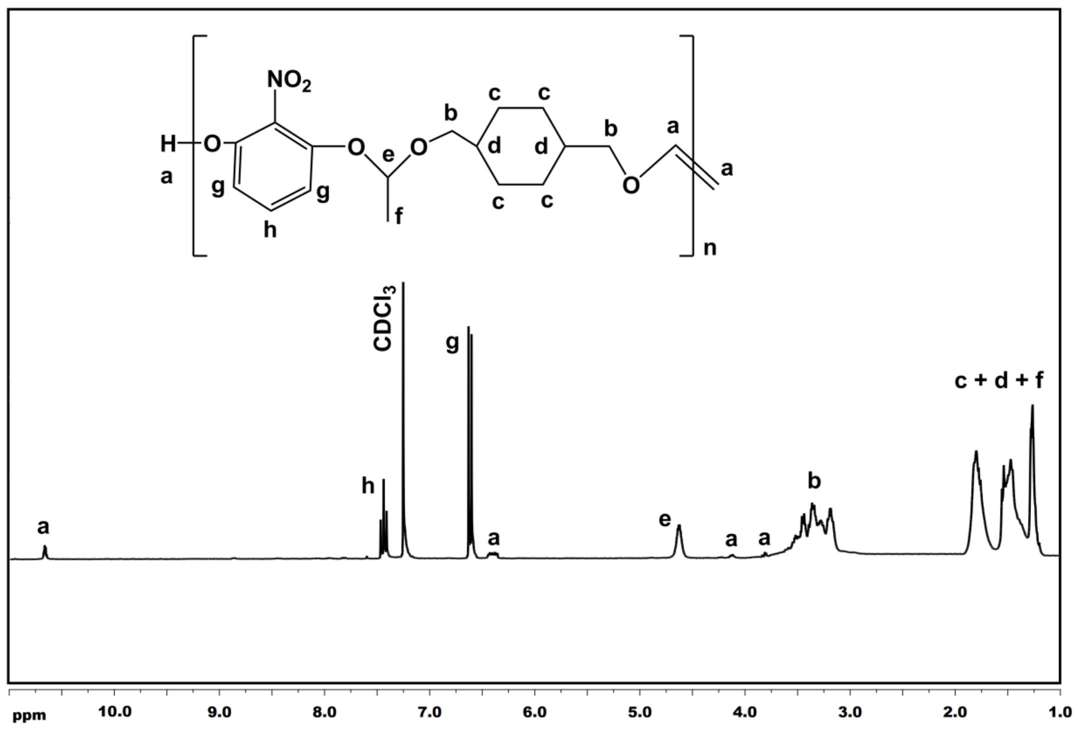

Supplementary Figure 1. ^1H NMR spectrum of the precursor polymer in DMSO.

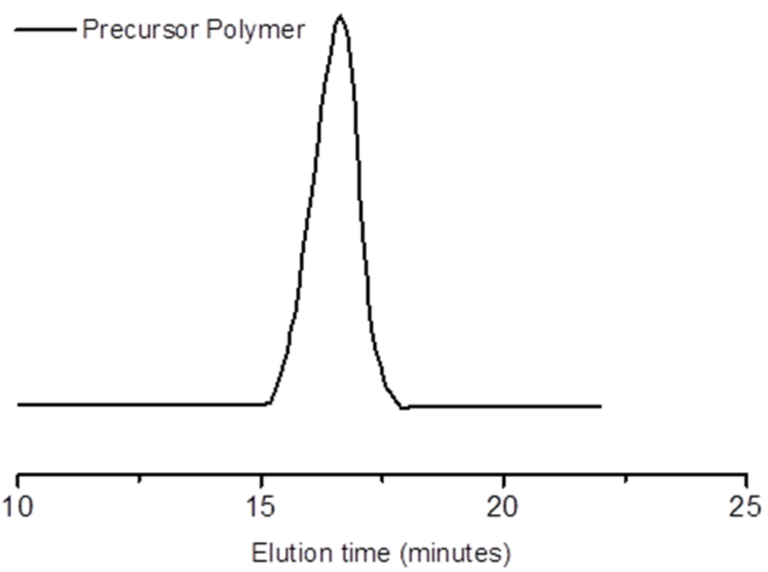

Supplementary Figure 2. GPC trace of the precursor polymer.

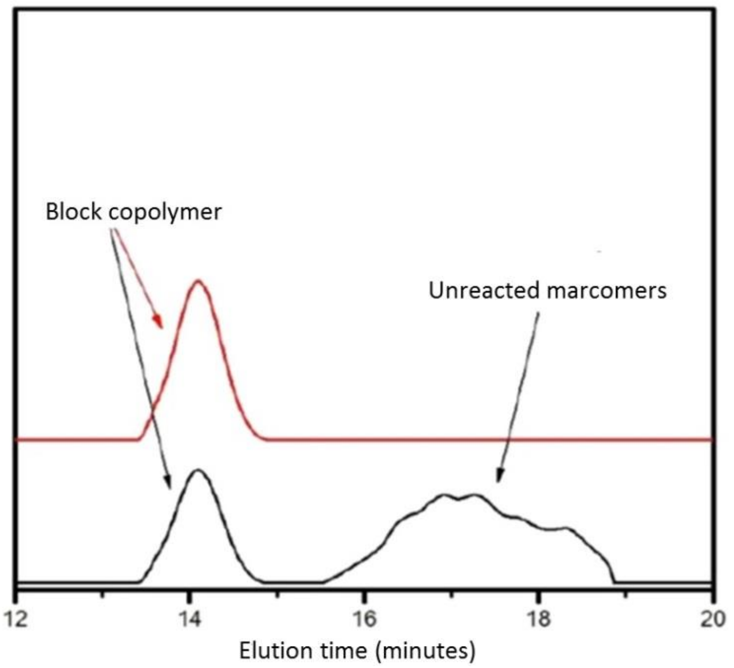

Supplementary Figure 3. GPC trace of the block copolymer before and after purification by dialysis.

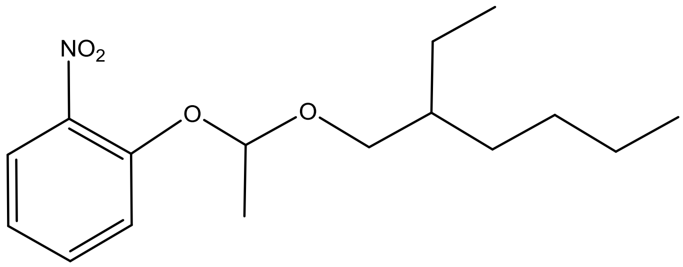

Supplementary Figure 4. Chemical structure of 2-nitrobenzene -2-ethylhexyl methyl acetal (Ac1).

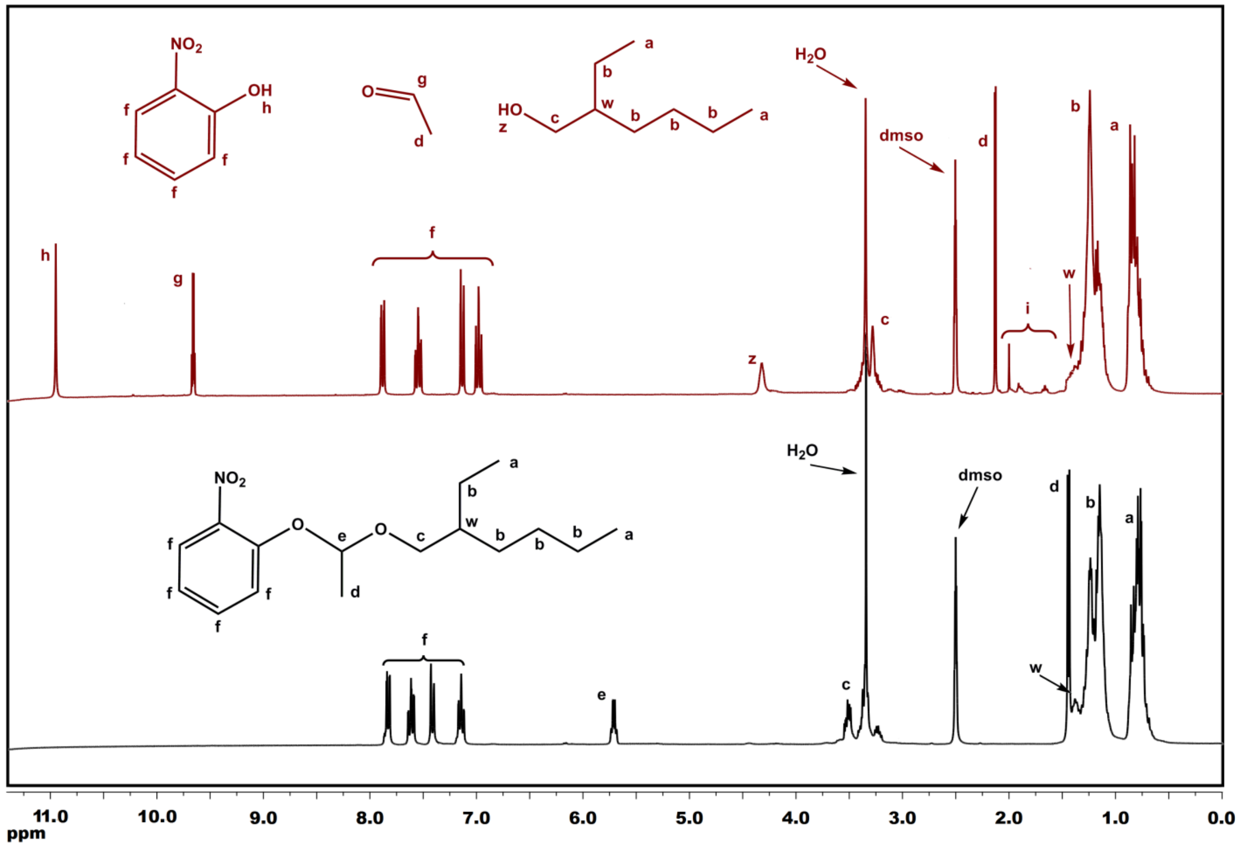

Supplementary Figure 5. ^{1}H NMR spectrum of Ac1 (bottom) and its photoproducts (top). Note the complete cleavage of the acetal bond (e), the formation of acetaldehyde (g and d), and the characteristic shifting of the methylene protons (c) adjacent to the hydroxyl formed moiety (z). In addition, the formation of carbonyl byproducts at lower concentrations is evidenced by (i) and (w) which is confirmed by the GC-MS data.

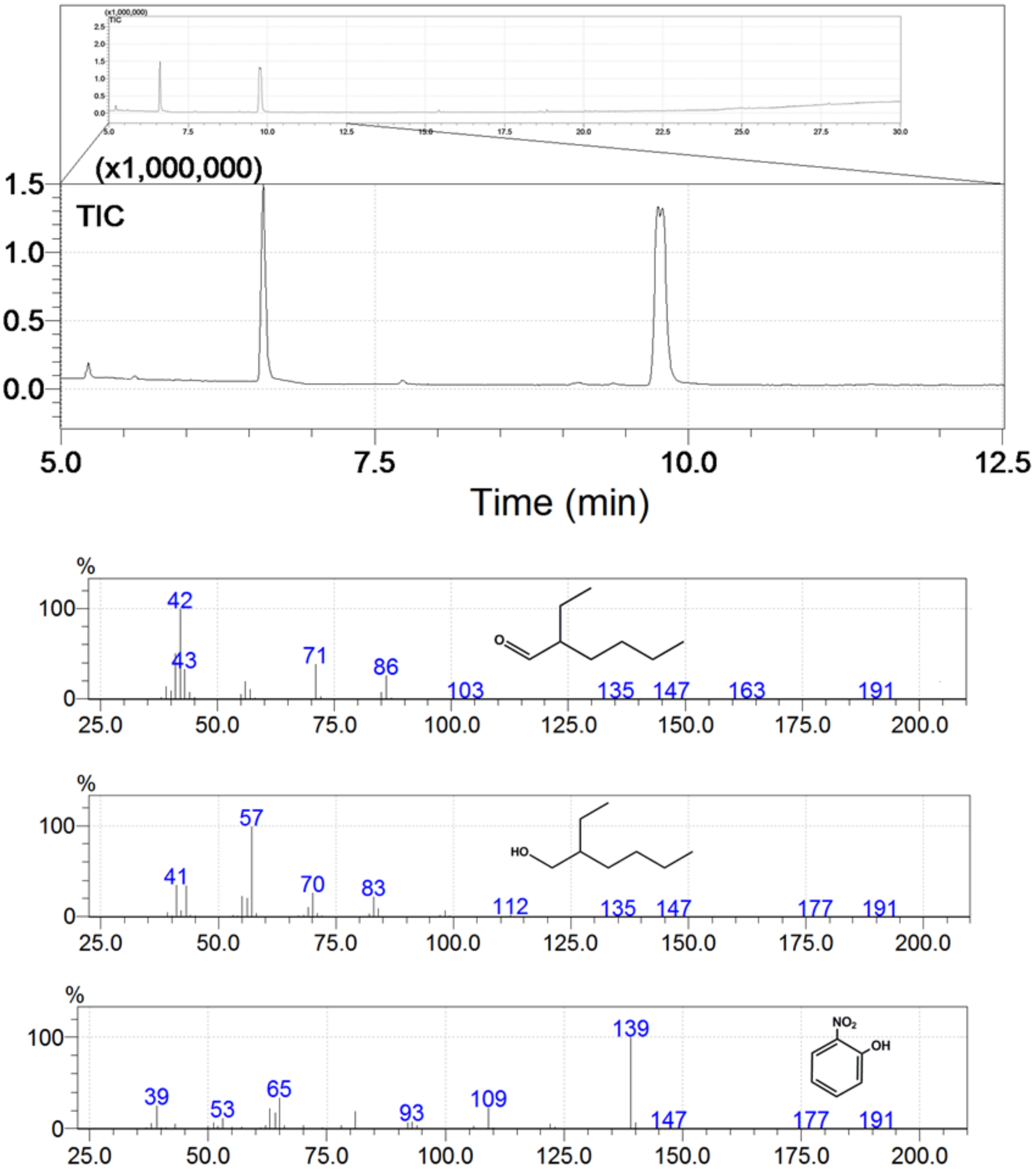

Supplementary Figure 6. GC-MS data of Ac1 photoproducts.

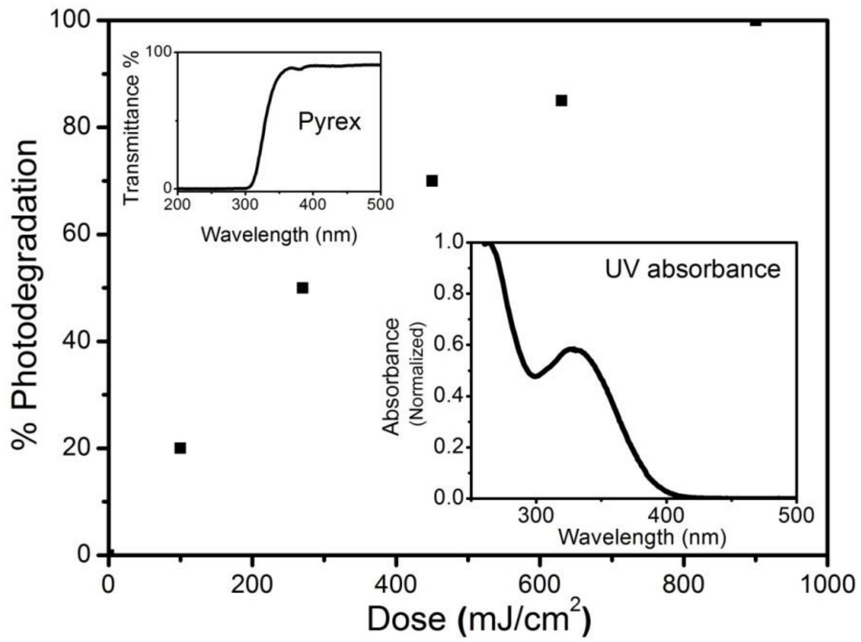

Supplementary Figure 7. Photolysis kinetics based on the irradiation of Ac1 at 365 nm derived by the gradual acetal proton peak diminution as observed by ^{1}H NMR. Inside diagrams show the transmittance profile of the pyrex filter used to cut off wavelengths below 300 nm (top left), and the full absorbance spectrum of Ac1 (bottom right).

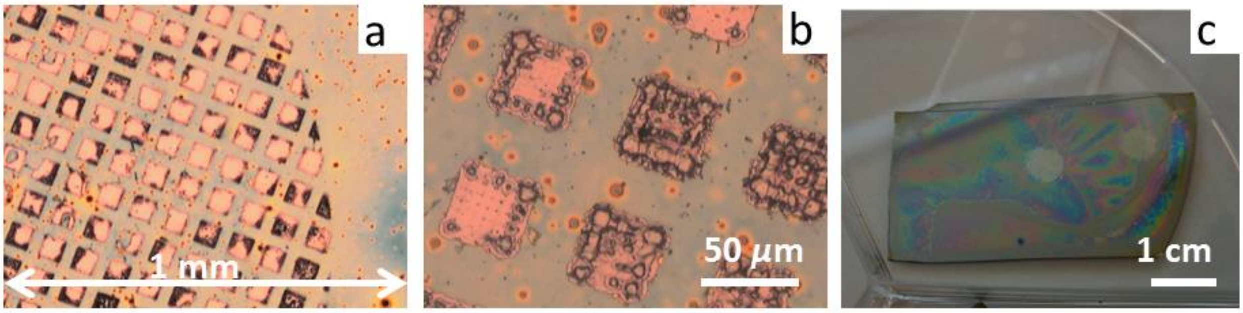

Supplementary Figure 8**.** Optical microscopy image of the pattern formation after laser ablation of the polymer film with a single pulse at 1064 nm (50 mJ/cm^2) by application of a TEM grid as photomask in a), close-up image of the Fresnel patterns formed in the squares of the photomask in b), and digital photograph of the ablated area from a polymer coated silicon wafer in c. Note the incomplete material removal (shown as darker areas in the squares as at the laser fluence is close to the ablation threshold.

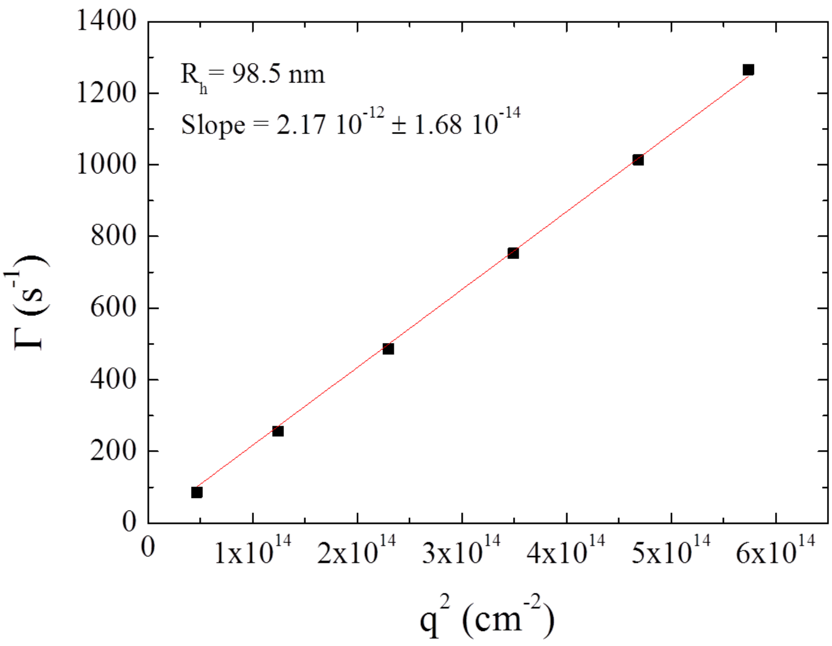

Supplementary Figure 9. Diagram derived from dynamic light scattering at different angles showing the linear increase of the decay rate as a function of the square wavevector from the slope of which the R_h is calculated.

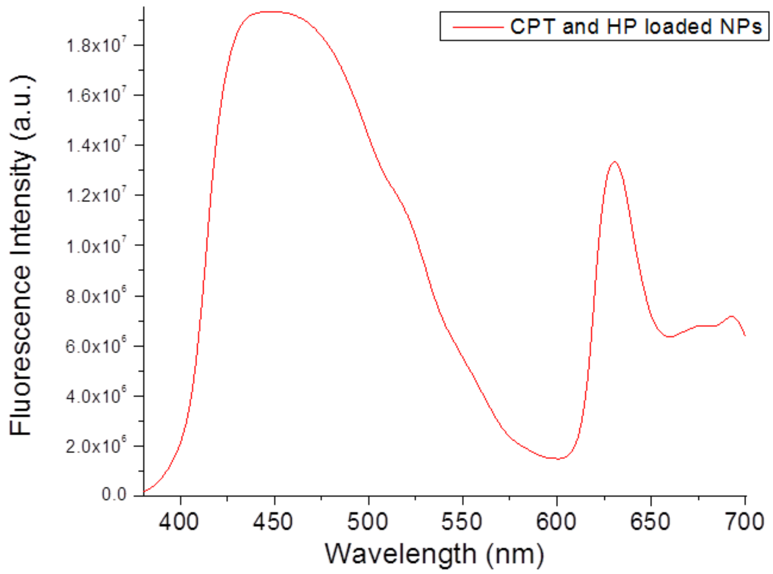

Supplementary Figure 10**.** Typical emission spectrum of drug loaded NPs in water. The excitation wavelength used was 350 nm where both CPT and HP are strong emitters allowing us to monitor the drug loading and release rates. Note the high emission intensity of CPT (at 450 nm) compared to HP (at 630 nm) which is attributed to the significantly higher quantum yield of the CPT molecule at this wavelength.

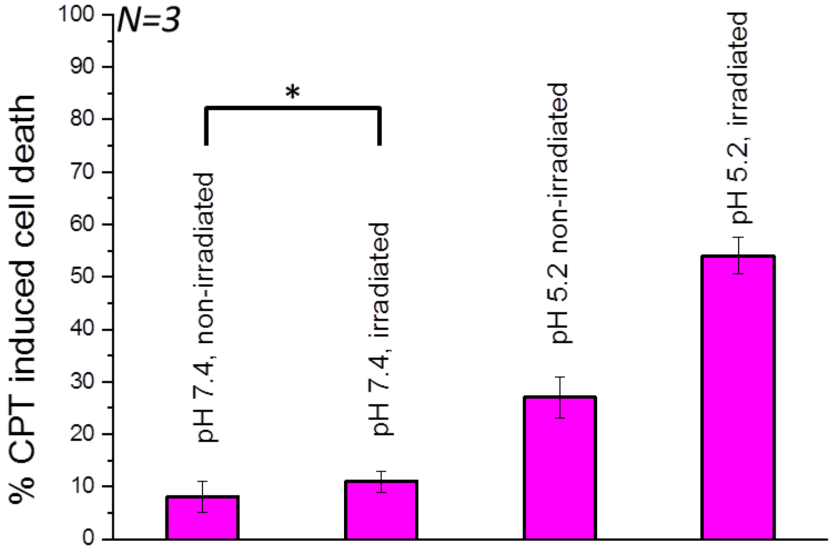

Supplementary Figure 11. The impact of CPT released after 4 hours (figure 3c) on HeLa cell death rates. * denotes statistically insignificant difference in the cell death rates of the alkaline samples.
